# Development of Pectin Particles as a Colon-Targeted Marjoram Phenolic Compound Delivery System

**DOI:** 10.3390/foods13020188

**Published:** 2024-01-06

**Authors:** María de las Nieves Siles-Sánchez, Paula García-Ponsoda, Irene Fernandez-Jalao, Laura Jaime, Susana Santoyo

**Affiliations:** Institute of Food Science and Research (CIAL), Universidad Autónoma de Madrid (CEI UAM+CSIC), 28049 Madrid, Spain; maria.siles@uam.es (M.d.l.N.S.-S.); paula.garcia@uam.es (P.G.-P.); irene.fernandez@inv.uam.es (I.F.-J.); laura.jaime@uam.es (L.J.)

**Keywords:** marjoram extract, encapsulation, nano/microparticles, pectin, phenolic compounds, colon delivery systems

## Abstract

Marjoram is a culinary herb that has been widely employed in folk medicine and presents a high content in phenolics. Thus, the aim of this project was to design formulations to encapsulate phenolic compounds from marjoram to allow their release in the colon. For this purpose, pectin was used as an encapsulating agent, applying two different encapsulation techniques (ionic gelation and spray-drying), followed by a CaCl_2_ bath. The ionic gelation technique showed a higher yield (77%) compared to spray-drying (31%), and the particles obtained were smaller (267 nm). However, the microparticles obtained by spray-drying presented a higher encapsulation efficiency (93%). Moreover, spray-dried microparticles protected a higher percentage of the encapsulated phenolics from the action of gastrointestinal pHs and enzymes. Hence, the results showed that spray-drying was a more appropriate technique than ionic gelation for the encapsulation of marjoram phenolics in order to protect them during the gastrointestinal step, facilitating their arrival in the colon. These microparticles would also be suitable for inclusion in food matrices for the development of phenolic colon delivery systems.

## 1. Introduction

The development of colon-specific oral delivery systems has become an important field of research. However, these systems must fulfil some requirements, such as avoiding the release of the incorporated therapeutic agents in the upper gastrointestinal tract after oral administration, and allowing their release when they reach the colon [1]. In this regard, several food biopolymers have been proposed as encapsulating agents. Among them, pectin plays an important role as an encapsulating material, since it is considered a GRAS food ingredient by the FDA; it also has the ability to remain resistant through the gastrointestinal tract, but is degraded by the enzymes produced by colonic microflora [2,3]. 

Pectin, a natural polysaccharide found on plant cell walls, consists of α-1→4-linked D-galacturonic acid units, which may be esterified with acetyl groups at C-2 or C-3. According to their degree of methyl-esterification, they are classified as high-methoxyl or low-methoxyl pectins [4]. In this regard, when the degree of methyl-esterification is higher than 50%, pectin can be classified as high methoxyl; meanwhile, when it is lower than 50%, it is named low-methoxyl [5]. Low-methoxyl pectin has been reported as an attractive candidate for colonic drug delivery systems, since it offers more resistance to pH changes than high-methoxyl pectin. In addition, low-methoxyl pectin is able to form gels in a wide pH range, in the presence of divalent cations, generally calcium (Ca^2+^). In these systems, gelation is produced due to the formation of intermolecular bonding zones between pairs of carboxyl groups in the smooth homogalacturonic regions of different chains in close contact, forming a structure called an ‘egg box’ [6].

In this context, recent studies have explored the potential of pectins, alone or in combination with other polymers, as an encapsulating agent for phenolic compounds [7]. With regard to encapsulation techniques using pectin as a wall material, ionic gelation has been described as an efficient and low-cost method that does not require specialized equipment, high temperatures, or organic solvents [8]. Meanwhile, spray-drying is a simple, continuous, versatile, and cost-effective process that allows the production of high-quality encapsulated products [9]. In this regard, Cai et al. [10] employed pectin for the encapsulation of curcumin through the preparation of calcium gel beads by ionic gelation. Likewise, Hu et al. [11] encapsulated flavonoids from *Satsuma mandarin* using the ionic gelation technique and pectin as an encapsulation polymer.

*Origanum majorana* L. (marjoram) represents one of the most widely used culinary and therapeutic herbs within the Lamiaceae family. This plant is native to the Mediterranean area, and it has been traditionally used in folk medicine for its efficacy in relieving various pathologies including gastrointestinal, respiratory, rheumatic, and neurological problems [2,12].

Extracts from marjoram have been reported to possess biological activities, including antioxidant, anti-inflammatory, antiproliferative, and antimicrobial [13]. In fact, these bioactivities have been mainly attributed to the presence of phenolic compounds such as rosmarinic acid, arbutin, lithospermic acid, or vicenin II, among others [14,15,16]. Moreover, these phenolics have been reported as prebiotic compounds due to their ability to modulate the composition of the gut microbiota [17,18,19,20].

However, it should be noted that phenolic compounds are unstable and susceptible to environmental factors, such as heat, light, oxygen, pH, and gastrointestinal enzymes. This leads to their easy degradation, and consequently, limits their biological activities such as the modulation of the gut microbiota [21]. Thus, their encapsulation is an important strategy in order to protect them during the gastrointestinal process, allowing their controlled release in the colon wherein they can exert their activity as prebiotic, interacting with the microbiota and consequently modulating it [22,23]. In this context, Tahir et al. [24] developed hesperidin colon-targeted nanoparticles. Similarly, Samprasit et al. [25] demonstrated the potential of chitosan nanoparticles containing α-mangostin and resveratrol as an oral encapsulation system against colon cancer. However, only a few studies have reported the encapsulation of phenolic compounds in plant extracts containing a wide range of such compounds for their delivery to the colon.

Therefore, the aim of this study was the design of pectin nano/microparticles loaded with a marjoram extract rich in phenolic compounds as colon delivery systems after oral administration. The protection of phenolics by these systems was evaluated by determining phenolic release profiles at gastrointestinal pHs, and after an in vitro gastrointestinal process. Moreover, the designed particles were suitable for inclusion in food matrices.

## 2. Materials and Methods

### 2.1. Reagents

Low-methoxyl pectin from citrus peel pectin and CaCl_2_ was purchased from Sigma-Aldrich (Madrid, Spain). Ethanol (99.5% purity), formic acid (99%), and acetonitrile of HPLC grade were from Panreac (Barcelona, Spain), Acros Organics (Madrid, Spain), and Macron Fine Chemicals (Madrid, Spain), respectively. Standard phenolic compounds (HPLC purity ≥ 95%) such as rosmarinic acid (≥98.5%) and eriodyctiol (≥98.9%) were from Sigma-Aldrich (Madrid, Spain). Lithospermic acid (≥ 98.8%), salvianolic acid (≥99.1%), apigenin (≥99.4%), apigenin 7-*O*-glucuronide (≥99.5%), and vicenin II (≥98.3%) were from Phytolab (Madrid, Spain). Ethyl gallate (≥99.1%), luteolin 7-*O*-glucuronide (≥95.2%), caffeic acid (≥99.9%), sterubin (≥98.0%), and luteolin 7-*O*-glucoside (≥98.9%) were obtained from Extrasynthese S.A. (Genay, France); arbutin (≥96.5%) was from TCI (Zwijndrecht, Belgium), and taxifolin (≥97.3%) from Fluka analytical (Madrid, Spain).

### 2.2. Plant Material and Ultrasound Assisted Extraction (UAE)

*Origanum majorana* L. dried leaves were purchased in Murciana Herboristería S.A (Murcia, Spain), and ground using a knife mill (Retsch Grindomix GM 200, Llanera, Spain) until a particle size of <500 µm was obtained. The UAE extraction followed the method described in Siles-Sánchez et al. [26]. Briefly, 20 g of ground leaves were extracted with ethanol (ratio of 1:10 plant:solvent) using a Branson 250 digital device (Branson Ultrasonic, Danbury, CT, USA) with an electric power of 200 W and frequency of 60 Hz. Then, the UAE extraction was carried out for 20 min at a 60% amplitude using a probe of ½″ diameter. Finally, samples were filtered, and ethanol was removed under a vacuum at 35 °C (IKA RV 10, Staufen, Germany). Dry extract was kept at −20 °C until its use.

### 2.3. Analysis of Phenolic Compounds (HPLC-PAD)

The analysis of the phenolic composition of the UAE extract was carried out using a high-performance liquid chromatography equipment (HPLC 1260 Infinity, Agilent Technologies, Santa Clara, CA, USA) with a photodiode array detector (PAD). The instrument was equipped with an ACE Excell 3 Super C18 reversed-phase column (Symta, Madrid, Spain) of 150 mm × 4.6 mm and a particle size of 3 µm, protected by a pre-column of the same material (10 mm × 3 mm). During the analysis, the temperature was kept at 35 °C with a sample injection of 20 µL. We followed the chromatographic method previously described by Villalva et al. [27], in which Milli-Q water with 1% formic acid and acetonitrile were used as phase A and B, respectively, keeping a flow rate of 0.5 mL/min. 

The identification of the compounds was carried out according to a previous study [28]. Calibration curves of authentic standards were used, where the hydroxy-methoxy flavones were quantified according to the calibration curve of apigenin. Ethyl gallate was employed as the internal standard in all samples.

### 2.4. Formation of Pectin Particles Loaded with Marjoram Extract

In this study, two different encapsulation techniques (ionic gelation and spray-drying) were performed for the encapsulation of the marjoram extract, using pectin as encapsulating agent.

#### 2.4.1. Ionic Gelation (IG)

Encapsulation of phenolic compounds using the ionic gelation technique was carried out in two different ways: following the protocol described by Sampaio et al. [29], and using a variant proposed by Hu et al. [11]. In both cases, different pectin and CaCl_2_ concentration solutions were prepared in Milli-Q water; meanwhile, the marjoram extract was dissolved in ethanol.

For the first protocol [29], 5 mL of the pectin solution (10 mg/mL) was mixed with 2 mL of the marjoram extract (1 mg/mL) under stirring until completely homogenized. This mixture was dripped using a syringe needle (23 G) at a distance of 20 cm into a CaCl_2_ water solution (20 mg/mL) under gentle agitation for 30 min to allow particle formation. Finally, the formed particles were obtained by centrifugation (Sorvall LYNX 6000 centrifuge, Thermo Scientific, MA, USA) at 13,000× *g* for 40 min.

For the second protocol [11], 2 mL of the different extract solutions (0.5, 0.75, 1 and 1.5 mg/mL) were mixed with 5 mL of the pectin solutions (1.25, 2.5, 3.75, 5 mg/mL) under gentle stirring at 50 °C for 1 h. Then, 1 mL of different CaCl_2_ solutions (6.25, 10 mM) were then added dropwise and left for 4 h under gentle stirring. After this time, the mixture was placed in a cold-water bath for 30 min, and finally centrifuged at 13,000× *g* for 40 min to obtain the formed microparticles.

The particles produced employing both protocols were freeze-dried (LyoBeta 15, Telstar, Madrid, Spain) and kept at −18 °C until use. In the same way, the supernatant volume produced after centrifugation was measured, and stored in refrigeration until use for the determination of the encapsulation efficiency.

#### 2.4.2. Spray-Drying (SP)

Encapsulation using spray-drying followed by a calcium chloride bath was carried out following the protocol of Lee et al. [30] with some modifications. In this case, different concentrations of pectin dissolved in water (10, 15, 20 and 25 mg/mL) and four different pectin–extract ratios (10:1, 8:1, 6:1 and 4:1) were tested. The marjoram extract was prepared in ethanol, keeping a concentration of 20 mg/mL. Prior to introducing the pectin–extract solution into the spray-dryer, the prepared solution of pectin and the necessary amount of marjoram extract were mixed until completely homogenized. After that, the different solutions were submitted to spray-drying (Büchi mini spray-dryer B-191, Barcelona, Spain) using the following working conditions: a feed rate of 4 mL/min, an inlet temperature of 170 °C, an aspiration rate 50%, a pump rate of 10%, and an air flow rate of 600 L/h. After spray-drying, 300 mg of the obtained particles were added to 20 mL of a 1 M CaCl_2_ solution under stirring for 10 min. Then, the mixture was centrifuged at 11,000 rpm for 10 min, and the particles were recovered and finally freeze-dried. 

### 2.5. Particles Characterization: Particle Size, Zeta Potential, and Polydispersity Index (PDI)

The obtained particles were characterized in terms of particle size, zeta potential, and PDI. For this purpose, a Zetasizer Ultra (Malvern Instruments Ltd., Malvern, UK) was employed. Briefly, determinations were carried out on particles dispersed in Milli-Q water using a homogenizer (Ultra-turrax T18 basic, IKA, Staufen, Germany) for 1 min. Measurements were made in triplicate for each sample.

### 2.6. Yield and Encapsulation Efficiency (EE) Determination

The process yields for both formulations were calculated as follows (Equation (1)):

(1) $$Yield\left(\%\right)=\left(\frac{weight\;of\;the\;micro/nanoparticles\;obtained}{weight\;of\;solids\;added\;to\;prepare\;the\;micro/nanoparticles}\right)\times 100$$

The encapsulation efficiency (EE) of the marjoram phenolic compounds encapsulated by ionic gelation was determined by HPLC-PAD in the supernatant recovered after particle centrifugation at 13,000× *g* for 40 min. For this purpose, prior to the analysis, the supernatants were recentrifuged using an Amicon filter 3 kDa (VWR, CA, USA) at 16,000× *g* for 45 min and filtered by 0.45 μm PVDF filter. Finally, the non-encapsulated compounds were determined, and the EE % was calculated using Equation (2). In addition, the individual EE (%) of each phenolic compound (individual EE) was also determined in the supernatant using Equation (3).

(2) $${EE}_{total}\left(\%\right)=100-\left(\frac{Ʃ\;supernatant\;phenolic\;compounds}{Ʃ\;phenolic\;compounds\;in\;extract}\right)\times 100$$

(3) $${EE}_{compound}\left(\%\right)=100-\left(\frac{Individual\;phenolic\;compound\;quantified\;in\;supernatant}{Individual\;phenolic\;compound\;quantified\;in\;extract}\right)\times 100$$

On the other hand, for the spray-dried particles, the encapsulation efficiency of the phenolic compounds was carried out as follows: 5 mL of Milli-Q water was added to 45 mg of the spray-dried particles, and shaken for 10 min. After that, 0.5 mL of the supernatant was filtered using an Amicon filter device as detailed before, then injected into the HPLC-PAD. Finally, the total and individual EE (%) were calculated according to Equations (2) and (3).

### 2.7. In Vitro Release Studies at Gastrointestinal pHs

The release of phenolic compounds from the formulated particles at gastrointestinal pHs was carried out following the protocol described by Cerchiara et al. [31], with some modifications. For this purpose, phosphate-buffered saline (PBS) at pH 2 and pH 7.4 was used, trying to simulate the pH of the stomach and intestine, respectively. The particles obtained by both techniques were processed in the same way. Briefly, 45 mg of particles were added to 5 mL of PBS and agitated. The mixture was left in a water bath (Memmert, WNE, Schwabach, Germany) at 37 °C with gentle agitation for 3 h, collecting 350 µL of the supernatant at 10 min, 1, 2 and 3 h. The aliquots were filtered using an Amicon filter device, as described before, prior to their injection into the HPLC. In this way, the quantity of phenolic compounds released during the test was determined. 

### 2.8. In Vitro Gastrointestinal Digestion

In vitro gastrointestinal digestion (oral, gastric, and intestinal) was reproduced using a Titrino Plus 877 (Methrom AG, Herisau, Switzerland), following the method described by Siles-Sánchez et al. [26].

Once the intestinal phase was completed, the sample was cooled to inactivate the enzymes and centrifuged at 8000 rpm for 15 min at 5 °C; the supernatant obtained was freeze-dried, then dissolved in DMSO and centrifuged with an Amicon 3 kDa filter device (VWR, CA, USA) (40 min at 16,000× *g*). Then, it was filtered with a 0.45 μm PVDF filter and analyzed by HPLC-PAD.

### 2.9. Statistical Analysis

Statistical analysis of the data obtained was performed using the Statgraphics Centurion version 19.4.01 (Statpoint Inc., Warrenton, VA, USA) software, subjecting them to a one-way analysis of variance (ANOVA) and using Fisher’s LSD (least significant difference) test for discrimination between means (*p* ≤ 0.05). The results obtained are expressed as the mean ± standard deviation.

## 3. Results and Discussion

### 3.1. Phenolic Characterization of UAE Marjoram Extract

The characterization of the phenolic composition of the marjoram extract was performed by HPLC-PAD. A total of 22 compounds were identified (Table 1), highlighting arbutin as the phenolic found in highest concentration, although rosmarinic acid (RA) was also presented in an important amount. The presence of other compounds such as lithospermic acid isomer and vicenin II in the extract was also remarkable. These results were consistent with previously reported studies [28,32].

### 3.2. Formulation Design and Particle Characterization: Ionic Gelation

The encapsulation of the marjoram extract was performed by ionic gelation (IG) using two different techniques, an established technique [29] and a variant of it [11]. In the traditional method, the mixture of pectin and extract was dripped over a CaCl_2_ bath, allowing cross-linking between the pectin and the divalent Ca^2+^ ion, which produces an “egg box” [33], and the subsequent formation of particles. When this method was employed, the developed particles presented a very large size (millimeters). Thus, since the aim of this work was to incorporate the formulated particles into foods, with the minimal change in their organoleptic properties, the particles produced by this technique were discarded. 

The variant proposed by Hu et al. [11] differed from the traditional method because CaCl_2_ was dripped onto a previously homogenized mixture of pectin and extract. Different concentrations of pectin, extract, and CaCl_2_ were tested to determine the optimal formulation. Formulation codes are shown in Table 2.

**Figure 1:**
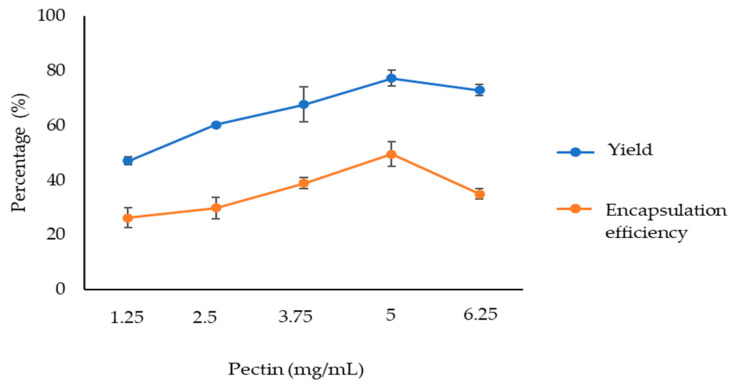
Yield and encapsulation efficiency (EE) of particles with different pectin concentrations (0.5 mg/mL extract and 6.25 mM CaCl_2_).

The first trial was to optimise the pectin concentration. Thus, the extract and CaCl_2_ concentrations were set at 0.5 mg/mL and 6.25 mM, respectively, varying the pectin concentration (formulations IG1, IG2, IG4, IG6 and IG10). The yield and the encapsulation efficiency (EE) values for the particles formulated are shown in Figure 1. As can be observed, the values of both parameters, yield and EE, increased with pectin concentration up to 5 mg/mL. Higher pectin concentrations showed lower values in both parameters. Thus, 5 mg/mL was determined as the optimum pectin concentration.

Regarding CaCl_2_, its concentration was increased to 10 mM, for a set extract concentration of 0.5 mg/mL, and testing pectin concentrations of 2.5, 3.75, 5 and 6.25 mg/mL (formulations IG3, IG5, IG7 and IG11, respectively). The results obtained, exposed no significant differences with respect to the formulations with 6.25 mM CaCl_2_. Therefore, the optimum CaCl_2_ concentration was set at 6.25 mM. These results were in concordance with those reported by Jung et al. [34]. These authors reported that CaCl_2_ concentration was not a significant parameter affecting indomethacin EE in the design of pectin hydrogels. 

Once the optimal concentration of pectin and CaCl_2_ was set at 5 mg/mL and 6.25 mM, respectively, the extract concentration was varied to 0.5, 0.75 and 1 mg/mL (formulations IG6, IG8 and IG9, respectively). Table 3 showed that no significant differences were found between using 0.5 or 0.75 mg/mL with respect to either yield or encapsulation efficiency. However, when using 1 mg/mL, a lower encapsulation efficiency was observed. In this context, Hu et al. [11] also indicated that for fixed pectin and CaCl_2_ concentrations, the EE increased with extract concentration up to a point. Beyond that point, the pectin concentration would not be enough to encapsulate all the added extract. Moreover, Yang et al. [9] reported that EE was influenced by the interactions between the extracts of phenolic compounds with the anionic pectin due to hydrogen bonds, and hydrophobic and electrostatic associations. Our EE values, ranging from 44.5 to 49.5 for IG8 and IG6, respectively, were similar to those reported by Bermúdez-Oria et al. [35] when using pectin for encapsulating phenolic antioxidants by ionic gelation. 

For this reason, the particle size, zeta potential and PDI were only determined for IG6 and IG8 formulations (Table 3). Regarding particle size, there were no significant differences between the nanoparticles obtained from both formulations (268.6 ± 2.5 to 275.4 ± 5.0 nm, respectively). This particle size was similar to that obtained by other authors [11] for citrus peel extract-loaded pectin nanoparticles. Furthermore, these formulated nanoparticles were stable, as zeta potential values were near −20 mV [36]. Moreover, values of the polydispersity index close to 0 indicated that the particle size was homogeneous, and that there were no aggregations between particles [36]. 

After these results, IG6 and IG8 formulations were selected for further studies.

### 3.3. Formulation Design and Particle Characterization: Spray-Drying

Previous assays were carried out to determine the optimal suspension viscosity, since too-viscous suspensions could block the spray-dryer and decrease the encapsulation yield [37]. Thus, several pectin solutions were prepared at 10, 15, 20 and 25 mg/mL, including the necessary extract quantity to achieve a pectin–extract ratio of 10:1. After carrying out these preliminary tests, it was concluded that the optimum concentration of pectin was 10 mg/mL, since the 15, 20 and 25 mg/mL solutions were too viscous. 

Once the optimal pectin concentration was determined, different pectin–extract ratios were tested (Table 4). As can be observed, only pectin–extract ratios of 10:1, 8:1 and 6:1 allowed the formation of particles, meanwhile, the 4:1 ratio did not produce any particles. 

Thus, the different formulations (SD1, SD2 and SD3) developed were subjected to several tests to determine the optimal formulation for the marjoram extract encapsulation. First, the yield and EE values were studied (Table 4). The SD1 formulation showed similar values for both yield and EE that SD2, but higher than those obtained from SD3. The yield value obtained in all cases was relatively low (≈30%) compared to the ionic gelation technique (≈80%). These lower yields have been frequently reported for microparticles prepared by spray-drying on laboratory scale because of the loss of the smaller particles during the encapsulation process [38].

However, the spray-drying technique showed higher EE values (>90%) than the ionic gelation technique (≈50%). These high EE values were not only the result of the strong interactions discussed above between phenolic compounds and pectin [9], but were also related to particle size. In this regard, it has been described that larger particle size corresponds to a higher extract loading capacity [10,39]. These results were in concordance with those obtained by Hartini et al. [40] that reported that the encapsulation of curcumin by spray-drying using different concentrations of pectin, obtaining EEs ranging from 69 to 92%.

The microparticles obtained (Table 4) presented sizes between 4.5 and 7.8 µm, with SD1 being the formulation with the higher particle sizes. As reported by Méndez et al. [41], particle size was related to the percentage of pectin and consequently to the amount of solids employed, obtaining larger particles when higher amounts of pectin were used [42]. In addition, as can be observed, the microparticles obtained by spray-drying presented a larger size compared to those obtained by ionic gelation. In this regard, Ozkan et al. [43] reported that during spray-drying, there is no control of droplet size and shape, leading to a wide range of size distribution, thus showing larger sizes.

The zeta potential of all the particles was similar, around −17 mV. These values indicated the high stability of the developed particles [36]. The PDI values obtained were closer to 1 than those obtained with ionic gelation, indicating the obtention of more heterogeneous particles [44]. These PDI values were again related to the lack of control over droplet size, meaning particles with a wide range of sizes were obtained [43].

Taking into account that SD1 particles presented the largest size and included the lowest quantity of extract, and EE values were similar to the SD2 and SD3 formulations, the SD1 formulation was discarded for further studies.

### 3.4. Encapsulation Efficiency (EE) of Different Yarrow Phenolics

Table 5 contains the EE values of phenolics for selected formulations (IG6, IG8, SD2 and SD3). Regarding formulations obtained by ionic gelation, the most polar compounds, such as arbutin or vicenin II, presented EE values between 34–39%, increasing as the polarity of the compounds decreases. Compounds with lower polarity, such as trihydroxy-trimethoxy flavone and sterubin, showed an EE between 66–83%. When comparing the two formulations, IG6 presented significantly higher EE values for most compounds than IG8 formulation. This fact could be related to the higher amount of extract included in the IG8 formulation. Thus, a part of the encapsulated extract could be weakly bound to the surface of the particles, being more easily released when particles were dispersed in water to calculate the EE values [38].

Formulations obtained using the spray-drying technique (SD2, SD3) also showed that as the polarity of the compounds decreased, their encapsulation efficiency increased. Thus, arbutin and vicenin II, the most polar compounds, presented the lowest encapsulation percentages (ranging from 80 to 91%). In increasing the phenolics’ polarity, the percentage of encapsulation increased to 100% for most compounds. When comparing the two formulations, the EE values were slightly higher for SD2 particles.

Considering these results, microparticles obtained using spray-drying presented higher EE values for all phenolics studied than ionic gelation formulations. Besides, between each technique, IG6 and SD2 also presented the higher EE values.

### 3.5. Controlled Released Studies at pH 2 and pH 7.4

In order to evaluate the influence of gastrointestinal pHs on the release of encapsulated phenolics, a release test was performed at pH 2 (simulating gastric pH) and at pH 7.4 (simulating intestinal pH). Release kinetics were performed at both pHs for the formulations IG6 and SD2, analyzing aliquots taken at 10 min, 1 h, 2 h and 3 h (Table 6). The results obtained at 1 and 2 h were quite similar to those acquired at 3 h, so in table, only values at 3 h are shown.

The release at 10 min would indicate the quantity of compounds that were located on/or very close to the surface of the particles, while the data at 3 h would indicate the release of the encapsulated compounds over this time. Thus, Casanova et al. [38] reported that a large initial burst may be due to the desorption of the compounds that had been bound to the encapsulating agent on the surface of the particles.

The IG6 formulation (Table 6) showed a significant release (around 60%) of arbutin and vicenin II at pH 2 after 10 min, remaining constant at 3 h, which would reinforce the idea that these compounds were on or very close to the surface of the nanoparticles formed. As the polarity of phenolics compounds decreased, the release of the compounds diminished, and from the lithospermic acid isomer onwards, no release was observed. However, it cannot be ruled out that the higher release of polar compounds could also be influenced by the affinity of these compounds to the medium used to carried out the release experiments, in this case PBS [45].

SD2 formulation presented a different release pattern, with a small initial release of arbutin and vicenin II after 10 min, indicating that the amount of these compounds on the surface of particles was low. After 3 h, the release of compounds increased, but never exceeded 12%. 

Results at pH 7.4 showed, in general, a similar release of compounds than at pH 2. Nevertheless, some compounds showed a higher release rate at pH 7.4. This fact could be related to the instability of pectin in an alkaline environment (pH 7.4), consequently resulting in a higher release of phenolics from the particles [46]. Thus, previous studies demonstrated similar release behavior. Jung et al. [34] observed a greater release of indomethacin included in pectin hydrogel beads at pH 7.4 in comparison to pH 2. In the same way, Hu et al. [11] reported a similar behavior for the release of flavonoids encapsulated using pectin.

Comparing the two encapsulation techniques, at both pHs, the release of the more polar compounds was higher when using ionic gelation.

### 3.6. Gastrointestinal Digestion

Finally, an in vitro gastrointestinal digestion was performed with the selected formulations (IG6 and SD2), in order to determine the influence of both pH and gastrointestinal enzymes on the developed formulations.

Table 7 showed the compounds detected after gastric and intestinal steps. The results also indicated that as the polarity of the compounds decreased, the quantity detected was lower. Regarding the IG6 formulation, arbutin and vicenin II were the compounds with the highest release rates (52–86%), while the rest of the compounds showed release rates below 20%. For the SD2 formulation, most phenolics remained encapsulated during gastrointestinal digestion, facilitating their arrival to the colon. Arbutin and vicenin II showed again the highest release rates, although less than 20%. It should be noted that the amount of phenolics released after gastrointestinal digestion was similar to that reported in the previous release kinetics, indicating almost no effect of gastrointestinal enzymes on the developed formulations. These data were in line with expectations, as pectin is a polysaccharide not digestible by enzymes in the upper gastrointestinal tract, so the presence of these enzymes should not produce the degradation of pectin and the subsequent release of the compounds [47]. Consequently, when the microparticles reach the colon, the pectin will be degraded by enzymes produced by the microbiota present in the colon, thereby releasing the phenolic compounds. However, inter-individual differences in the microbiota must be taken into account, as these could affect the pectin degradation, and hence the release of phenolic compounds.

Comparing the results for the two formulations, it could be concluded that the spray-dried particles protected phenolics in a higher way throughout the digestion, allowing a higher percentage of these compounds to reach the colon. 

## 4. Conclusions

The two techniques proposed in this work, ionic gelation and spray-drying, were suitable for the formation of pectin particles loaded with marjoram extract. The ionic gelation technique produced stable nanoparticles with yield values closer to 80% and encapsulation efficiency values around 50%. Meanwhile, microparticles obtained by spray-drying presented a lower yield value (30%), but a higher encapsulation efficiency (90%). Regarding the individual phenolic compounds, the encapsulation efficiency increases with decreasing polarity, regardless of the encapsulation technique used. 

The release profile for different phenolics at both gastrointestinal pHs showed that most of them remained inside the microparticles, with a higher release for those with a more polar character. Besides, the results after the in vitro gastrointestinal digestion were quite similar to those reported in the release studies, indicating that the enzymes did not degrade the formulated particles. 

Comparing the two formulations, spray-drying microparticles allowed for the encapsulation and delivery to the colon of a higher amount of marjoram phenolics, suggesting this technique is more suitable than ionic gelation for the design of colonic delivery systems including phenolics.

However, colonic fermentation studies would be necessary to ensure that the pectin will be degraded by the microbiota, hence allowing the release of polyphenols in the colon.

## Figures and Tables

**Table 1:** Characterization and quantification of the phenolic compounds of the UAE marjoram extract by HPLC-PAD. Data shown are expressed as mg compound/g dry sample and represent mean ± S.D (*n* = 3).

Peak	Compound	Concentration (mg/g)
1	Arbutin	84.34 ± 0.89
2	Luteolin hexoside-pentoside	0.20 ± 0.01
3	Vicenin II	4.56 ± 0.07
4	Caffeic acid	0.47 ± 0.00
5	Orientin	0.42 ± 0.01
6	6-hydroxyluteolin 7-*O*-glucoside	0.76 ± 0.01
7	Luteolin 7-*O*-glucoside	1.06 ± 0.06
8	Luteolin 7-*O*-glucuronide	3.27 ± 0.06
9	Taxifolin	2.33 ± 0.02
10	Apigenin 7-*O*-glucuronide	2.24 ± 0.03
11	Rosmarinic acid	48.96 ± 0.46
12	Lithospermic acid isomer	8.48 ± 0.07
13	Salvianolic acid isomer	2.09 ± 0.06
14	Eriodyctiol	1.26 ± 0.01
15	Luteolin	0.21 ± 0.00
16	Trihydroxy-methoxy flavone	1.78 ± 0.00
17	Trihydroxy-dimethoxy flavone I	1.34 ± 0.02
18	Trihydroxy-dimethoxy flavone II	1.02 ± 0.01
19	Naringenin	0.34 ± 0.00
20	Apigenin	0.21 ± 0.00
21	Trihydroxy-trimethoxy flavone	1.29 ± 0.02
22	Sterubin	3.51 ± 0.02

**Table 2:** Formulation conditions in ionic gelation (IG) assays.

Code	Pectin (mg/mL)	Extract (mg/mL)	CaCl_2_ (mM)
IG1	1.25	0.5	6.25
IG2	2.5	0.5	6.25
IG3	2.5	0.5	10
IG4	3.75	0.5	6.25
IG5	3.75	0.5	10
IG6	5	0.5	6.25
IG7	5	0.5	10
IG8	5	0.75	6.25
IG9	5	1	6.25
IG10	6.25	0.5	6.25
IG11	6.25	0.5	10

**Table 3:** Characterization of nanoparticles obtained by ionic gelation (mean ± SD, *n* = 3). EE (encapsulation efficiency), PDI (polydispersity index).

Code	Pectin (mg/mL)	Extract (mg/mL)	Yield (%)	EE (%)	Mean Particle Size (nm)	Zeta Potential (mV)	PDI
IG6	5	0.50	77.3 ± 2.9 ^a^	49.5 ± 4.5 ^a^	268.6 ± 2.5 ^a^	−21.7 ± 3.0 ^a^	0.31 ± 0.06 ^a^
IG8	5	0.75	79.2 ± 5.5 ^a^	44.5 ± 4.1 ^a^	275.4 ± 5.0 ^a^	−22.0 ± 1.0 ^a^	0.33 ± 0.03 ^a^
IG9	5	1.00	77.1 ± 0.5 ^a^	24.7 ± 2.0 ^b^	-	-	-

^a,b^ Different superscript letters denote significant differences within the same column (*p* ≤ 0.05).

**Table 4:** Characterization of nanoparticles obtained by spray-drying (SD) (mean ± SD, *n* = 3). Relation Pec–Ext (relationship of pectin–extract), EE (encapsulation efficiency), PDI (polydispersity index).

Code	Pectin (mg/mL)	Extract (mg/mL)	Relation Pec–Ext	Yield (%)	EE (%)	Particle Size (µm)	Zeta Potential (mV)	PDI
SD1	10	1.00	10:1	36.9 ± 1.4 ^a^	96.8 ± 1.6 ^a^	7.8 ± 0.5 ^a^	−15.8 ± 0.7 ^a^	0.68 ± 0.07 ^a^
SD2	10	1.25	8:1	31.4 ± 0.9 ^b^	93.4 ± 1.3 ^b^	5.9 ± 0.5 ^b^	−16.5 ± 0.4 ^a^	0.73 ± 0.25 ^a^
SD3	10	1.67	6:1	28.7 ± 3.3 ^b^	90.6 ± 0.3 ^c^	4.5 ± 1.0 ^b^	−17.3 ± 1.5 ^a^	0.78 ± 0.19 ^a^
SD4	10	2.50	4:1	-	-	-	-	-

^a,b,c^ Different superscript letters denote significant differences within the same column (*p* ≤ 0.05).

**Table 5:** Encapsulation efficiency (%) of different phenolic compounds in formulations IG6, IG8, SD2 and SD3 (mean ± SD, *n* = 3).

Compound	Ionic Gelation		Spray-Drying	
	IG6	IG8	SD2	SD3
Arbutin	39.1 ± 3.8 ^a^	36.0 ± 3.6 ^a^	90.8 ± 1.2 ^a^	87.8 ± 0.1 ^b^
Vicenin II	36.3 ± 3.6 ^a^	34.2 ± 5.8 ^a^	84.3 ± 1.9 ^a^	79.5 ± 1.2 ^b^
Taxifolin	48.4 ± 3.3 ^a^	45.4 ± 3.4 ^a^	100.0 ± 0.0 ^a^	91.3 ± 3.7 ^b^
Apigenin 7-*O*-glucuronide	45.3 ± 3.3 ^a^	40.4 ± 1.9 ^a^	100.0 ± 0.0 ^a^	91.1 ± 0.6 ^b^
Rosmarinic acid	57.4 ± 2.4 ^a^	50.6 ± 2.4 ^b^	95.1 ± 3.1 ^a^	92.6 ± 0.6 ^a^
Lithospermic acid isomer	83.4 ± 1.9 ^a^	79.9 ± 4.0 ^a^	100.0 ± 0.0 ^a^	100.0 ± 0.0 ^a^
Salvianolic acid isomer	81.0 ± 1.2 ^a^	75.1 ± 2.0 ^b^	100.0 ± 0.0 ^a^	100.0 ± 0.0 ^a^
Eriodyctiol	72.9 ± 4.5 ^a^	57.9 ± 2.5 ^b^	100.0 ± 0.0 ^a^	100.0 ± 0.0 ^a^
Trihydroxy-dimethoxy flavone I	73.6 ± 3.3 ^a^	62.4 ± 1.1 ^b^	100.0 ± 0.0 ^a^	100.0 ± 0.0 ^a^
Trihydroxy-trimethoxy flavone	75.4 ± 2.1 ^a^	66.3 ± 2.7 ^b^	100.0 ± 0.0 ^a^	100.0 ± 0.0 ^a^
Sterubin	83.3 ± 3.2 ^a^	74.7 ± 5.1 ^b^	100.0 ± 0.0 ^a^	100.0 ± 0.0 ^a^

^a,b^ Different superscripts letters denote significant differences within lines obtained with the same encapsulated technique (*p* ≤ 0.05).

**Table 6:** Amount (%) of phenolic compounds released after 10 min and 3 h from IG6 and SD2 at pH 2 (A) and pH 7.4 (B).

**(A)**				
** Compound **	**Release 10 min (%)**		**Release 3 h (%)**	
	**IG6**	**SD2**	**IG6**	**SD2**
Arbutin	57.9 ± 5.0 ^a^	5.2 ± 0.5 ^b^	62.3 ± 3.1 ^a^	6.5 ± 0.1 ^a^
Vicenin II	53.9 ± 5.7 ^a^	10.1 ± 0.4 ^a^	59.5 ± 6.7 ^a^	11.1 ± 2.0 ^a^
Taxifolin	34.1 ± 3.4 ^a^	0.0 ± 0.0 ^b^	38.2 ± 4.8 ^a^	3.9 ± 0.9 ^a^
Apigenin 7-*O*-glucuronide	6.7 ± 1.5 ^a^	0.0 ± 0.0 ^b^	9.1 ± 2.4 ^a^	3.0 ± 0.0 ^a^
Rosmarinic acid	4.9 ± 1.4 ^a^	0.9 ± 0.3 ^a^	4.5 ± 1.7 ^a^	1.4 ± 0.7 ^a^
Lithospermic acid isomer	0.0 ± 0.0 ^a^	0.0 ± 0.0 ^a^	0.0 ± 0.0 ^a^	0.0 ± 0.0 ^a^
Salvianolic acid isomer	0.0 ± 0.0 ^a^	0.0 ± 0.0 ^a^	0.0 ± 0.0 ^a^	0.0 ± 0.0 ^a^
Eriodyctiol	0.0 ± 0.0 ^a^	0.0 ± 0.0 ^a^	0.0 ± 0.0 ^a^	0.0 ± 0.0 ^a^
Trihydroxy-dimethoxy flavone I	0.0 ± 0.0 ^a^	0.0 ± 0.0 ^a^	0.0 ± 0.0 ^a^	0.0 ± 0.0 ^a^
Trihydroxy-trimethoxy flavone	0.0 ± 0.0 ^a^	0.0 ± 0.0 ^a^	0.0 ± 0.0 ^a^	0.0 ± 0.0 ^a^
Sterubin	0.0 ± 0.0 ^a^	0.0 ± 0.0 ^a^	0.0 ± 0.0 ^a^	0.0 ± 0.0 ^a^
**(B)**				
** Compound **	**Release 10 min (%)**		**Release 3 h (%)**	
	**IG6**	**SD2**	**IG6**	**SD2**
Arbutin	54.3 ± 3.7 ^b^	6.3 ± 1.9 ^a^	69.6 ± 3.0 ^a^	6.6 ± 0.9 ^a^
Vicenin II	64.0 ± 6.8 ^a^	10.7 ± 3.4 ^a^	73.9 ± 5.2 ^a^	14.5 ± 0.1 ^a^
Taxifolin	30.0 ± 2.8 ^b^	0.0 ± 0.0 ^b^	48.1 ± 4.8 ^a^	5.0 ± 0.0 ^a^
Apigenin 7-*O*-glucuronide	7.2 ± 2.5 ^a^	0.9 ± 0.3 ^b^	11.8 ± 2.9 ^a^	1.5 ± 0.2 ^a^
Rosmarinic acid	15.1 ± 2.9 ^a^	6.3 ± 1.3 ^a^	9.9 ± 2.0 ^b^	5.1 ± 0.7 ^a^
Lithospermic acid isomer	0.0 ± 0.0 ^a^	0.0 ± 0.0 ^a^	0.0 ± 0.0 ^a^	0.0 ± 0.0 ^a^
Salvianolic acid isomer	0.0 ± 0.0 ^a^	0.0 ± 0.0 ^a^	0.0 ± 0.0 ^a^	0.0 ± 0.0 ^a^
Eriodyctiol	0.0 ± 0.0 ^a^	0.0 ± 0.0 ^a^	0.0 ± 0.0 ^a^	0.0 ± 0.0 ^a^
Trihydroxy-dimethoxy flavone I	0.0 ± 0.0 ^a^	0.0 ± 0.0 ^a^	0.0 ± 0.0 ^a^	0.0 ± 0.0 ^a^
Trihydroxy-trimethoxy flavone	0.0 ± 0.0 ^a^	0.0 ± 0.0 ^a^	0.0 ± 0.0 ^a^	0.0 ± 0.0 ^a^
Sterubin	0.0 ± 0.0 ^a^	0.0 ± 0.0 ^a^	0.0 ± 0.0 ^a^	0.0 ± 0.0 ^a^

^a,b^ Different superscripts letters denote significant differences within lines obtained at the same time (*p* ≤ 0.05).

**Table 7:** Phenolic compounds (%) found after gastrointestinal digestion of IG6 and SD2.

Compound	Gastric Step (%)		Intestinal Step (%)	
	IG6	SD2	IG6	SD2
Arbutin	52.0 ± 5.9	6.6 ± 1.7	60.2 ± 8.7	18.9 ± 0.1
Vicenin II	86.8 ± 1.4	18.3 ± 2.9	74.1 ± 9.0	17.8 ± 0.0
Taxifolin	12.4 ± 3.6	2.3 ± 0.9	16.9 ± 2.8	8.8 ± 0.9
Apigenin 7-*O*-glucuronide	3.2 ± 1.1	6.4 ± 2.4	8.4 ± 1.2	5.6 ± 1.5
Rosmarinic acid	6.1 ± 0.6	8.6 ± 1.7	21.3 ± 4.2	10.5 ± 1.3
Lithospermic acid isomer	0.0 ± 0.0	0.0 ± 0.0	0.0 ± 0.0	0.0 ± 0.0
Salvianolic acid isomer	0.0 ± 0.0	0.0 ± 0.0	0.0 ± 0.0	0.0 ± 0.0
Eriodyctiol	0.0 ± 0.0	0.0 ± 0.0	0.0 ± 0.0	0.0 ± 0.0
Trihydroxy-dimethoxy flavone I	0.0 ± 0.0	0.0 ± 0.0	0.0 ± 0.0	0.0 ± 0.0
Trihydroxy-trimethoxy flavone	0.0 ± 0.0	0.0 ± 0.0	0.0 ± 0.0	0.0 ± 0.0
Sterubin	0.0 ± 0.0	0.0 ± 0.0	0.0 ± 0.0	0.0 ± 0.0

## Data Availability

Data are contained within the article.
